# Temperature and Estrogen Alter Predator–Prey Interactions between Fish Species

**DOI:** 10.1093/iob/obaa008

**Published:** 2020-04-01

**Authors:** J L Ward, V Korn, A N Auxier, H L Schoenfuss

**Affiliations:** 1 Department of Biology, Ball State University, 2111 W Riverside Ave, Muncie, IN 47306, USA; 2 Aquatic Toxicology Laboratory, Department of Biology, St. Cloud State University, 720 4th Avenue South, St Cloud, MN 56301, USA

## Abstract

A variety of environmental estrogens are commonly detected in human-impacted waterways. Although much is known about the effects of these environmental estrogens on the reproductive physiology and behavior of individuals within species, comparatively less is known about how these compounds alter the outcomes of interactions between species. Furthermore, few studies have considered how the effects of contaminants are modulated by natural variation in abiotic factors, such as temperature. To help fill this knowledge gap, we conducted a factorial experiment to examine the independent and combined effects of estrone (E1) and temperature on the outcome of predator–prey interactions between two common North American freshwater fishes, fathead minnows (*Pimephales promelas*) and bluegill sunfish (*Lepomis macrochirus*). Larval fathead minnows and adult sunfish were exposed to either a low (mean±standard deviation, 90.1 ± 18 ng/L; *n* = 16) or high (414 ± 147 ng/L; *n* = 15) concentration of E1 or to a solvent control for 30 days at one of four natural seasonal temperatures (15°C, 18°C, 21°C, and 24°C) before predation trials were performed. Exposure to E1 was associated with a significant increase in larval predation mortality that was independent of temperature. Across all temperature treatments, approximately 74% of control minnows survived; this survivorship significantly exceeded that of minnows exposed to either concentration of E1 (49% and 53% for minnows exposed to the low and high concentrations, respectively). However, exposure to E1 also impaired the prey-capture success of sunfish, partially mitigating predation pressure on exposed minnows. Overall prey-capture success by sunfish showed an inverted U-shaped distribution with temperature, with maximal prey consumption occurring at 21°C. This study illustrates the vulnerability of organismal interactions to estrogenic pollutants and highlights the need to include food web interactions in assessments of risk.

## Introduction

A variety of anthropogenic stressors are known to induce changes in the behavior of resident wildlife in human-dominated aquatic systems, including increases in temperature, nutrient enrichment and reductions in water clarity, and urban and agricultural chemical influxes (Giusi et al. 2005; Tuomainen and Candolin 2011; Hayden et al. 2015; Cook et al. 2018; Hasan et al. 2018). For example, increased turbidity alters the intensity and spectral composition of light (Collins and Hart 2015) and can degrade the transmission of visual stimuli, leading to changes in behavioral responses (Seehausen et al. 1997; Candolin et al. 2007; Glotzbecker et al. 2015). Other forms of anthropogenic environmental change, such as chemical contaminants, have unique potential to disrupt normal behavior by changing internal physiological function, motivation, or stimulus processing (Weis and Khan 1991; Faucher et al. 2008). Among the best-studied are estrogenic contaminants that disrupt endocrine function (Jobling and Tyler 2003; Clotfelter et al. 2004); in fish, exposure to endocrine disrupting chemicals (EDCs) has been shown to alter behavior in a variety of contexts, including social and sexual behavior, foraging, and predation (see for example, reviews by Sloman and McNeil 2012; Söffker and Tyler 2012; Saaristo et al. 2018).

One way that EDCs can alter the dynamics of populations and communities under pressure is by impairing the ability of aquatic organisms to perceive, recognize, or appropriately respond to biotic stimuli (e.g., Dell’Omo 2002; Sloman and Wilson 2006; Fabian et al. 2007; Munday et al. 2011; Ward and Blum 2012; Halfwerk and Slabbekoorn 2015). For example, male fishes exposed to environmental estrogens show reduced courtship effort and levels of aggression (Colman et al. 2009) and female fishes show changes in mate choice (Coe et al. 2008; Saaristo et al. 2009). However, whereas many studies have examined the effects of EDCs on reproductive function and behavior within single species, comparatively less is known about how these compounds alter the outcomes of interactions between species, such as in the case of predation where both the predator and prey may be exposed (Weis and Candelmo 2012).

The complexity of biotic and abiotic factors that influence ecological communities can make it difficult to generalize the effects of contaminants to the outcomes of predator–prey interactions (Baudrot et al. 2018). Species may differ in their responsiveness to contaminants, making the extrapolation of results from one species to another problematic (Lange et al. 2012). Contaminant-induced behavioral alterations can reduce the ability of exposed individuals to react to predators, either by impairing sensory systems that are important for the detection of potential threats (Scholz et al. 2000; Faucher et al. 2008; Munday et al. 2011; Ehrsam et al. 2016) or inducing changes in locomotor responses; for example, by increasing reaction time or decreasing the speed of escape (McGee et al. 2009; Painter et al. 2009; Ward et al. 2017). Exposure may also promote risky behaviors that increase vulnerability to predation (Bell 2004; Nakayama et al. 2005; Brodin et al. 2013; Heintz et al. 2015). However, contaminants that impair prey responses may also impair predator feeding behavior by affecting motivation to feed, or reducing search effectiveness or the ability to capture prey (Brown et al. 1987; Weis and Khan 1991; Smith et al. 1995), potentially mitigating increased vulnerability of prey species (Weis and Khan 1990; Grippo and Heath 2003).

Moreover, naturally varying abiotic factors, such as temperature, pH, or salinity, can also modulate effects of contaminants on organismal responses (Cairns et al. 1975; Hall and Anderson, 1995; Gordon 2003; Körner et al. 2008; Pelletier et al. 2006; Laskowski et al. 2010; Lange et al. 2018), or combine with life-history traits to confer increased vulnerability at specific developmental stages or in particular seasons (Liney et al. 2005; Salice et al. 2011; Przeslawski et al. 2015; Lange et al. 2018). Few studies have explored how variation in the ambient environment might impact the effects of exposure on complex species interactions (Hayden et al. 2015). Temperature in particular is a key factor regulating developmental, physiological, and metabolic processes in fish (Beitinger et al. 2000; Pankhurst and Munday 2011) and natural seasonal variation in ambient temperature therefore has significant potential to modulate contaminant-induced changes in predator–prey interactions. However, predators and prey may differ in their responses to such fluctuations (Stenseth et al. 2002; Freitas et al. 2007; Grigaltchik et al. 2012) because thermal sensitivities can vary between species (Johnston and Temple 2002; Guderley 2004).

In this study, we investigated the effects of a common urban environmental estrogen, estrone (E1) (Ankley et al. 2017) on the outcomes of predator–prey interactions between a common forage fish, larval fathead minnow (*Pimephales promelas*), and piscivorous sunfish (*Lepomis macrochirus*) across a range of temperatures reflective of natural spring and summer variation. E1 is a natural estrogen that is excreted by females in wastes and is one of the most prevalent steroid hormones in human-impacted aquatic systems (Barber et al. 2012; Ma et al. 2016; Adeel et al. 2017). Although E1 is often considered to pose a lower ecological risk due to its reduced potency compared with other environmental estrogens, recent research suggests that the potential impact of E1 on individuals, populations, and communities may be underestimated (Ankley et al. 2017). Notably, exposure to E1 has been shown to impair anti-predator behavior in larval fish (McGee et al. 2009; Ward et al. 2017) suggesting that it has the potential to alter predator–prey dynamics (Rearick et al. 2018); however, whether such changes translate into higher predation rates and how E1 affects predator efficacy is unknown.

## Materials and methods

### Experimental design

To test the hypothesis that chronic, low-dose exposure to E1 across a range of temperatures alters predator–prey interactions, we separately but simultaneously exposed adult bluegill sunfish and larval fathead minnows to either a low or high concentration of E1 (i.e., E1_low_ or E1_high_), or to an equivalent volumetric percentage of solvent (100% ethanol [EtOH]) at one of four temperatures (15°C, 18°C, 21°C, or 24°C) for 30 days. The temperature range used in the experiment approximated the range of natural spring and summer variation in northern waterways and was well within the thermal tolerance limits for *L. macrochirus* (Stuber et al. 1982) and *P. promelas* (Pyron and Betiinger 1993). Water quality parameters, including pH, temperature (°C), and dissolved oxygen (mg/L), were monitored on a daily basis using a handheld multi-parameter sampling instrument (model 556 MPS, YSI Instruments, OH, USA). On day 30, we conducted predation trials to examine the effects of temperature and estrogenic exposure on larval predation risk and the prey-capture effectiveness of predatory sunfish. All subjects were sacrificed at the conclusion of the experiment via an overdose of NaCO_2_-buffered MS-222 (Western Chemical, WA, USA). The Institutional Animal Care and Use Committee at St. Cloud State University, St Cloud, MN, approved all procedures and maintenance protocols used in the experiments (protocol number 8-73).

### Exposure chemicals

Powdered E1 (≥99% purity, Sigma–Aldrich, St. Louis, MO) was dissolved in 100% EtOH to create stock solutions and stored in 1 mL aliquots at −20°C for the duration of the experiment. Aqueous exposure solutions with nominal concentrations of 125 and 625 ng/L for the E1_low_ and E1_high_ treatments, respectively, were prepared every 3 days in darkened glass carboys by adding an appropriate amount of the stock to 10 L of conditioned well water. An aqueous control treatment was also prepared that contained an equivalent volumetric percentage of solvent (0.0002% v/v EtOH). Previous studies have reported no effect of the EtOH solvent at similar or higher concentrations (Schoenfuss et al. 2002; Jorgenson et al. 2015); therefore, it is unlikely that exposure to the solvent affected our observations. Concentrations of E1 used in the study were selected for consistency with previous work (Ward et al. 2017; Cox et al. 2018) and because they fall within the environmental range of estradiol equivalency quotients (EEQs) reported in the literature (Kolpin et al. 2002; Martinović et al. 2007); the E1 low concentration in particular represented a high environmentally relevant concentration of E1, and had a total estrogenic activity similar to EEQ values previously reported for North America and Europe; for example, Martinović et al. (2007) measured an EEQ of 44 ng/L (approximately 400 ng/L E1 equivalent at a 10:1 activity ratio of E2 to E1) in wastewater effluent in northern Minnesota. Similarly, Elliott et al. (2017) calculated EEQs in tributaries of the Great Lakes as high as 28 ng/L (280 ng/L E1 equivalent). Water samples from Venice Lagoon were reported to have estrogenic activities ranging from 1.1 to 191 ng/L EEQ (∼2000 ng/L E1 equivalents; Pojana et al. 2007). Aqueous exposure solutions were thoroughly mixed by agitating the carboys for 10 s before tightly covering the necks of the carboy with aluminum foil. Water samples were taken at regular intervals throughout the experiment and frozen at −20°C for LC–MS/MS analysis of chemical concentration (Schultz et al. 2013).

### Exposure regime and apparatus

#### Sunfish

Adult bluegill sunfish were obtained from 10,000 Lakes Aquaculture (Osakis, MN) and treated with *Fungal Cure* (API Fishcare). The sunfish were exposed to E1_low_, E1_high_, or the EtOH control treatment for 30 days at 15°C, 18°C, 21°C, or 24°C under flow-through conditions (Zhao et al. 2017). Subjects were maintained for the duration of the experiment in 52-L aquaria (15 fish per aquarium) under a 16:8 h light:dark cycle. Two aquaria were used for each concentration of E1 and four aquaria were used for control subjects. Subjects were fed using a mixture of blood worms and brine shrimp *ad libitum* twice daily. In addition, sunfish were periodically offered live minnows to condition them to the novel food source.

#### Minnow larvae

Minnow larvae (1-day post-hatch [dph]; Environmental Consulting and Testing, Superior, WI) were randomly assigned to 1-L glass jars (Ball Corp.) containing either E1_low_, E1_high_, or the solvent control (∼30 minnows per jar) and maintained for 30 days under a 50% daily static renewal protocol and a 16:8 h light:dark cycle. Each day, half of the water in each jar was removed and replaced with fresh E1-treated water or control water taken directly from the flow-through exposure lines that fed the sunfish tanks. This ensured that the same water was used for both minnows and sunfish exposures, but that the minnows were not subjected to chemical cues of the predator. As appropriate to the treatment, water temperature was maintained at 15°C, 18°C, 21°C, or 24°C throughout the exposure period using water baths or heating pads. Minnows were fed freshly hatched brine shrimp *ad libitum* twice daily, beginning 2 dph.

#### Florescent staining

Subjects from either the control or the exposed group were marked 1 day before use in a behavioral trial using a fluorescent SE-MARK calcein dye (Western Chemical, Ferndale, WA, USA) according to approved US Food and Drug Association Investigational New Animal Drug protocols (FDA INAD 10-987). The group selected to undergo staining in each trial was randomly determined to prevent mark-associated bias. Larvae from the control, E1_low_, and E1_high_ treatments were maintained for 6 h in separate stain baths created by adding a 1.0% calcein stock solution to conditioned well water until a concentration of 250 mg/L was reached. Preliminary trials confirmed that florescence persisted until the end of the experiment and no abnormal behaviors were observed during staining. The larvae recovered from each trial were identified using a SE-MARK detector to illuminate fluorescently marked fish.

### Predation trials

We conducted a semi-factorial behavioral predation experiment on day 30 that paired control and exposed larvae in a competitive setting, thereby permitting direct estimates of increased predation mortality due to exposure (16 total exposure scenarios; Table 1). Our target number of trials was 20 per scenario; however, we were interested in relative survival ratios and therefore excluded trials in which the predator ate 0% or 100% of the larvae. Our final dataset included 6–19 trials per exposure scenario. Trials were conducted in opaque-walled PVC arenas with a 104-cm diameter and a water depth of 25 cm (total volume: 212 L). Twenty evenly spaced artificial plants were added to each arena to provide refuge for the larvae. Trials were conducted at 15°C, 18°C, 21°C, or 24°C (±1°C), as appropriate to the exposure treatment. The arena was drained and scrubbed between trials to remove any residual chemical cues.

**Table 1:** Sixteen exposure scenarios for competitive predation trials that paired control larval fathead minnows against minnows exposed to E1_low_ or E1_high_ in the presence of a non-exposed or exposed piscivore at four temperatures (15°C, 18°C, 21°C, and 24°C**)**

	Trial exposure scenario		
Temperature (°C)	Predator	Prey (control)	Prey (exposed)
15	E1_low_	Control	E1_low_
15	E1_high_	Control	E1_high_
15	Control	Control	E1_low_
15	Control	Control	E1_high_
18	E1_low_	Control	E1_low_
18	E1_high_	Control	E1_high_
18	Control	Control	E1_low_
18	Control	Control	E1_high_
21	E1_low_	Control	E1_low_
21	E1_high_	Control	E1_high_
21	Control	Control	E1_low_
21	Control	Control	E1_high_
24	E1_low_	Control	E1_low_
24	E1_high_	Control	E1_high_
24	Control	Control	E1_low_
24	Control	Control	E1_high_

Focal sunfish were fasted for 72 h before being used in the experiment to maximize motivation to forage. At the start of a trial, one sunfish (control, E1_low_, or E1_high_) was placed in the arena and given approximately 1.5 h to acclimate. At the end of the acclimation period, one group of control larvae and one group of exposed larvae were simultaneously introduced to the arena. Trials conducted at 18°C, 21°C, and 24°C paired five exposed and five control larvae (10 total larvae); trials conducted at 15°C paired four exposed and four control minnows, respectively, due to the availability of individuals. In each trial, the focal sunfish was permitted to forage for larvae for 1 h, after which the test was stopped and the sunfish removed via a hand net. Surviving larvae were immediately captured and transferred to a glass beaker for identification of group assignment (see the section “Fluorescent staining”). We assessed predation upon exposed versus control larval subjects by comparing the relative proportion of exposed versus control minnows in each trial that survived.

### Statistics

We compared the survival of sunfish and larval minnows, respectively, during the exposure period using chi-square tests. Differences in larval growth (body length [BL], measured on day 21) across treatments were tested via analysis of variance (ANOVA).

To assess the effect of exposure on larval survival versus controls, we compared the percent survival of paired exposed and non-exposed minnows in each of the 16 different trial scenarios using Wilcoxon signed-rank tests. To evaluate the independent and interactive effects of temperature, and predator and prey exposure levels on capture success, we calculated the average proportion of total larvae consumed in each trial [(exposed + control)/2] and compared the proportion of larvae consumed via an ANOVA, with temperature (15°C, 18°C, 21°C, and 24°C), predator exposure level (control, E1_low_, E1_high_), prey exposure level (E1_low_, E1_high_), and both predator and prey exposure level×temperature interactions specified as fixed effects. The dependent variable was arc sin-transformed prior to analyses to satisfy parametric assumptions.

## Results

### Water quality

Measured E1 concentrations (mean±SD) were 90 ± 18 and 414 ± 147 ng/L for the E1_low_ (*n* = 16 samples) and E1_high_ (*n* = 15) treatments, respectively. E1 was not detected in control samples. Water temperatures throughout the exposure period were 16.2 ± 1.1°C, 18.3 ± 0.6°C, 21.8 ± 0.4°C, and 24.1 ± 0.6°C, for the 15°C, 18°C, 21°C, and 24°C treatments, respectively. Water quality in the exposure tanks also remained stable throughout the experiment (dissolved oxygen=5.5 ± 0.9 mg/L; pH=8.3 ± 0.2).

### Larval survival and growth

Neither survival nor growth during the exposure period was affected by E1 concentration or temperature for minnow larvae and sunfish. Survival at day 30 was high and consistent across treatments, ranging from (mean±SD) 85.5 ± 13.2% to 89.5 ± 8.7% for larvae and 88.3 ± 3.7% to 93.5 ± 11.1% for sunfish. Chi-square tests revealed no differences in survival among treatments for either species (*P*s >0.05). Larval BL on day 21 was also similar among the 12 treatments, ranging from mean±SD of 7.69 ± 1.48 to 8.89 ± 1.22 mm. An ANOVA revealed no significant effect of temperature or concentration level, or an interaction between the two factors on growth (overall model: *F*_11,170_=0.880, *P* = 0.561).

### Predation trials

**Fig. 1:**
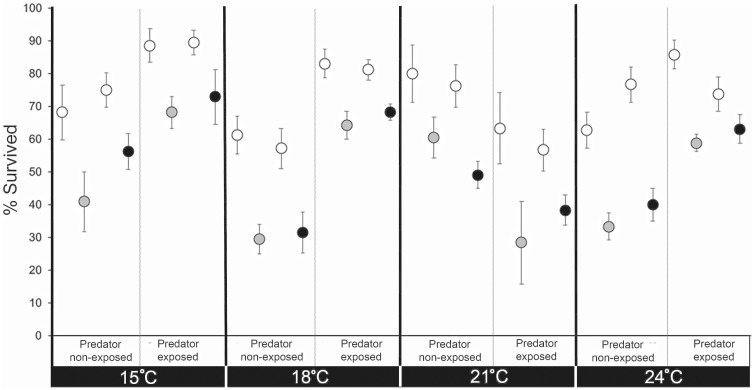
Percent survival of larval fathead minnows in competitive predation trials. Larvae were exposed to either a low (E1_low_; gray symbols) or high (E1_high_; black symbols) dose of E1 for 30 days or to an equivalent volumetric percentage of solvent (control; white symbols) at one of four temperatures (15°C, 18°C, 21°C, and 24°C**)**. Points and whiskers are mean±SEM.

A total of 219 sunfish and 2,096 minnow larvae were used in the predation trials (*n* = 6–19 trials per exposure scenario; Table 2). Across all temperature treatments, the mean±SD survival of control, E1_low_, and E1_high_ larvae was 74 ± 23%, 49 ± 24%, and 53 ± 23%, respectively. In all 16 exposure scenarios, exposed larvae were more likely to suffer predation compared with controls; this difference was statistically significant in 14 of 16 test combinations (Fig. 1 and Table 2). Exceptions to this finding occurred only at 24°C (predator: E1_high_, prey: E1_high_) and 21°C (predator: control, prey: E1_low_). In these tests, there was no statistical difference in the survival of paired control versus exposed fish.

**Table 2:** Wilcoxon signed-rank tests comparing the percent survival of larval fathead minnows exposed to either E1_low_ or E1_high_ for 30 days with that of non-exposed larvae (control) in predation trials conducted at four temperatures (15°C, 18°C, 21°C, and 24°C**)**

	Treatment			Wilcoxon test	
	Predator	Prey	*N*	*Z*	*P*
15°C	Control	Low	11	−2.52	**0.012**
	Control	High	12	−2.18	**0.029**
	Low	Low	11	−2.71	**0.007**
	High	High	12	−2.06	**0.039**
18°C	Control	Low	17	−3.46	**0.001**
	Control	High	14	−3.45	**0.001**
	Low	Low	19	−2.69	**0.007**
	High	High	17	−2.29	**0.022**
21°C	Control	Low	9	−1.37	0.169
	Control	High	11	−2.59	**0.009**
	Low	Low	6	−2.02	**0.043**
	High	High	12	−2.48	**0.013**
24°C	Control	Low	15	−3.37	**0.001**
	Control	High	12	−3.12	**0.002**
	Low	Low	17	−3.16	**0.002**
	High	High	13	−1.51	0.131

Significant effects are given in bold.

**Fig. 2:**
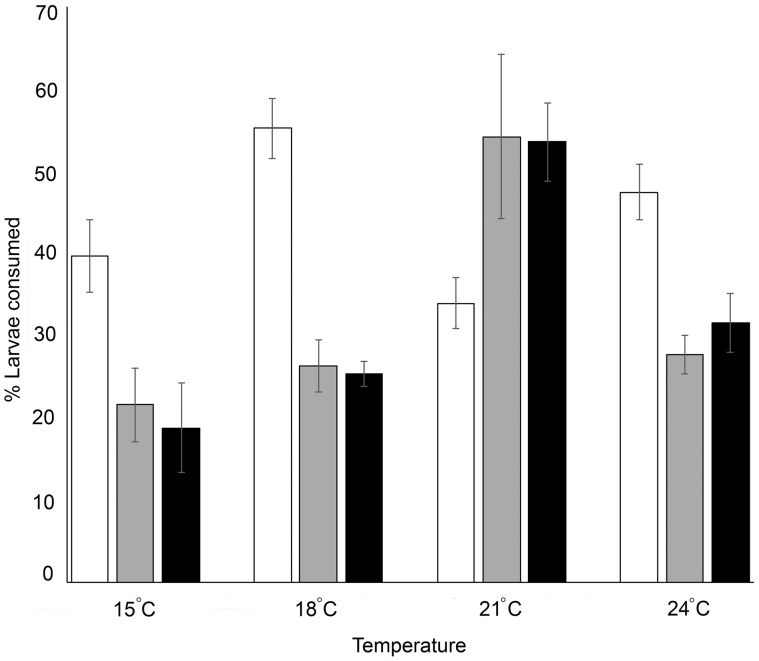
Overall prey-capture success of predatory sunfish (% of total larvae in the trial consumed). Sunfish were exposed to either a low (E1_low_; gray bars) or high (E1_high_; black bars) dose of E1 for 30 days or to an equivalent volumetric percentage of solvent (control; white bars). Bars and whiskers represent the mean±SEM.

An ANOVA indicated that exposure of the predator to E1 had a significant effect on prey consumption (*F*_2,192_=10.82, *P* < 0.001). Pairwise *post hoc* tests (least significant difference; LSD) indicated that prey consumption was negatively associated with predator exposure (Fig. 2); at most of the temperatures tested, non-exposed sunfish successfully captured and consumed more larvae than sunfish exposed to E1_low_ (*P* < 0.001) or E1_high_ (*P* = 0.003). An exception to this finding occurred at 21°C, where sunfish exposed to E1_low_ and E1_high_ consumed more larvae than control subjects (temperature×predator exposure interaction: *F*_6,192_=8.99, *P* < 0.001; Fig. 2). There was no difference in the proportion of larvae eaten in trials that exposed the predator to E1_low_ or E1_high_ (*P* = 0.58). Temperature also had a significant overall effect on prey consumption (*F*_3,192_=7.47, *P* < 0.001). Across the tested temperature range, prey consumption increased from 15°C to 21°C (15°C versus 18°C: *P* = 0.013; 18°C versus 21°C: *P* = 0.005) before decreasing slightly at 24°C (21°C versus 24°C: *P* = 0.003).

We did not find a significant effect of prey exposure level on overall prey consumption (*F*_1,192_=1.07, *P* = 0.30), indicating that both doses of E1 had a similar effect on prey. We also did not find a significant temperature×prey exposure interaction (*F*_3,192_=1.82, *P* = 0.15), indicating that the mortality of larvae exposed to E1_low_ or E1_high_ did not differ depending on temperature.

## Discussion

In this study, we examined the extent to which ambient temperature and exposure to E1 modified the outcomes of predator–prey interactions between two fish species. Across a 9°C temperature range reflective of natural spring and summer temperature fluctuations, exposure to E1 reduced the overall prey-capture success of piscivorous sunfish but increased the likelihood of predation upon exposed larval minnows. These data suggest that in addition to altering reproductive physiology and behavior, E1 has significant potential to disrupt ecological interactions between predators and prey and alter the structure and function of food webs in aquatic communities (Nilsen et al. 2019).

Compared with control fish, predatory *L. macrochirus* exposed to E1 had a 14–16% reduction in prey-capture success across most of the range of temperatures tested; an exception to this pattern occurred at 21°C, where the reverse pattern occurred. Notwithstanding this exception, our findings are consistent with previous studies reporting impaired foraging success following exposure to a variety of contaminants (Weis et al. 2001), including EDCs **(**Hallgren et al. 2014). For example, Weis et al. (2003) reported that prey-capture success by larval mummichogs (*Fundulus heteroclitus*) was negatively related to the concentrations of several contaminants in sediments. Hallgren et al. (2014) similarly reported a significant reduction in the foraging success of EE2-exposed roach (*Rutilus rutilus*) upon *Daphnia magna* and an associated reduction in biomass, likely as a result of lower food intake. Reductions in foraging success following exposure to aquatic contaminants have been variously attributed to sensory and neural changes in the perception and recognition of prey stimuli, impaired locomotion responses, and reduced motivation to forage (Brown et al. 1987; Weis and Khan 1991; Smith et al. 1995; Saaristo et al. 2018). Additional studies are now needed to differentiate among these alternative mechanisms.

Despite the fact that exposure to E1 reduced overall predation upon experimental populations, exposed larvae were disproportionately consumed within most trial scenarios; specifically, *P. promelas* larvae exposed to E1 suffered 22–25% higher predation mortality compared with control larvae across the range of natural spring and summer temperatures tested. Increased predation mortality following exposure to environmental contaminants has been reported in a number of aquatic species (Weis et al. 2001), including *P. promelas*; for example, Rearick et al. (2018) showed that the survival of larvae exposed to low or high concentrations of 17β-estradiol was reduced by 10% and 11%, respectively, in predation trials involving sunfish. However, that study only considered the effects of exposure on prey. Our results thus extend those of Rearick et al. (2018) by investigating the outcomes of predator–prey interactions in cases where both the predator and the prey are exposed, across a range of temperatures.

Considered as a whole, the results of this study suggest that exposure to environmental estrogens in anthropomorphized environments has a disproportionate impact on prey during predator–prey interactions, similar to that reported for other classes of contaminants. McIntyre et al. (2012) reported that brief exposure to copper levels of 5–20 µg/L eliminated behavioral alarm responses in coho prey, causing increased detection, reduced evasion, and increased mortality. In another study, copper also significantly reduced both dragonfly (predator) and tadpole (prey) activity levels, but the effect on tadpoles was much larger than on dragonflies, the latter of which was heavily influenced by temperature (Hayden et al. 2015). Notably, the magnitudes of reductions in both the foraging success of *L. macrochirus* and survival of *P. promelas* in this study were similar at both levels of exposure (E1_low_ and E1_high_); this finding is important because it suggests that the adverse effects of exposure on the behavioral responses of predators and prey may follow a non-dose-dependent model and that the lower effect threshold for predation survival effects has yet to be determined.

Predator–prey interactions in our study were also significantly affected by thermal regime. Temperature is the most important environmental variable influencing the metabolism and physiology of fish and other ectotherms (Clark and Johnston 1999). It has been shown to influence predation in various fish species (Persson 1986) by altering the number and/or kinematics of predator attacks or the escape performance of prey (Grigaltchik et al. 2012; Allan et al. 2015). Similar to patterns observed in other species (e.g., Biro et al. 2007), overall prey consumption in our study by sunfish showed an inverted U-shaped distribution with temperature across trial scenarios, with consumption increasing from 15°C to a maximum at 21°C before declining slightly at 24°C. These data are consistent with data showing that thermal swimming performance curve for sunfish peak at approximately 21–24°C (Jones et al. 2008), with increased thermal sensitivities above and below this value. However, we did not find that temperature modulated the effects of exposure on prey to influence predation mortality. Other studies investigating how temperature influences exposure-induced behavioral alterations have shown that not only can contaminants and temperature independently affect escape performance and mortality rates, but that the effects of contaminants can fluctuate with temperature (Janssens et al. 2014; Ward et al. 2017). For example, in a previous study, *P. promelas* exposed to E1 at the highest temperature studied here (24°C) showed antipredator escape-response latencies that were on average 18% longer and swimming speeds that were 50% slower than those of control fish (Ward et al. 2017). At present, it is unknown whether changes in the performance of predators or prey contribute more strongly to the outcomes of predator–prey interactions reported here. Behavioral performance studies of both predators and prey are now needed to fully understand how temperature and exposure interact to influence the outcome of predatory interactions.

## Conclusion

Whereas many studies have investigated the effects of EDCs on individual behavior, comparatively less is known about the effects of contaminants on ecological function or species interactions, despite the fact that these compounds have significant potential to disrupt communities (Clotfelter et al. 2004; Richmond et al. 2017). For example, Kidd et al. (2007, 2014) showed the collapse of a fathead minnow (*P. promelas*) population in an experimental lake following chronic exposure to low concentrations of a synthetic estrogen, 17-alpha ethynylestradiol was associated with both increased abundance of zooplankton and invertebrate species and a decline in the abundance of predatory lake trout (*Salvelinus namaycush*). The results of this study suggest that E1, prevalent in human-impacted aquatic systems, has the potential to alter species interactions similar to other classes of contaminants and that furthermore, the effects of exposure on both predators and prey can influence outcomes in unpredictable ways. Additional studies of species interaction that incorporate the effects of EDCs on both species *and* relevant abiotic factors are key to assessing population and community vulnerability in urban-impacted ecosystems.

## Funding

This research was supported by the Minnesota Environment and Natural Resources Trust Fund as recommended by the Legislative-Citizen Commission on Minnesota Resources [M.L. 2014, Chp. 226, Sec. 2, Subd. 03d.].
